# Heart Rate Variability-Based Subjective Physical Fatigue Assessment

**DOI:** 10.3390/s22093199

**Published:** 2022-04-21

**Authors:** Zhiqiang Ni, Fangmin Sun, Ye Li

**Affiliations:** 1 Joint Engineering Research Center for Health Big Data Intelligent Analysis Technology, Shenzhen Institute of Advanced Technology, Chinese Academy of Sciences, Shenzhen 518055, China; zq.ni@siat.ac.cn (Z.N.); fm.sun@siat.ac.cn (F.S.); 2 University of Chinese Academy of Sciences, Beijing 100049, China

**Keywords:** heart rate variability, physical fatigue, feature selection, machine learning

## Abstract

Accurate assessment of physical fatigue is crucial to preventing physical injury caused by excessive exercise, overtraining during daily exercise and professional sports training. However, as a subjective feeling of an individual, physical fatigue is difficult for others to objectively evaluate. Heart rate variability (HRV), which is derived from electrocardiograms (ECG) and controlled by the autonomic nervous system, has been demonstrated to be a promising indicator for physical fatigue estimation. In this paper, we propose a novel method for the automatic and objective classification of physical fatigue based on HRV. First, a total of 24 HRV features were calculated. Then, a feature selection method was proposed to remove useless features that have a low correlation with physical fatigue and redundant features that have a high correlation with the selected features. After feature selection, the best 11 features were selected and were finally used for physical fatigue classifying. Four machine learning algorithms were trained to classify fatigue using the selected features. The experimental results indicate that the model trained using the selected 11 features could classify physical fatigue with high accuracy. More importantly, these selected features could provide important information regarding the identification of physical fatigue.

## 1. Introduction

With the improvement of human living standards, more and more people realize the importance of exercise to health and engage in regular physical exercise to keep healthy. However, proper exercise is good for health, while excessive exercise may bring harm to the body(e.g., muscular and skeletal injuries [1], overtraining syndrome [2], atrial fibrillation [3] and immune function reduction). Excessive exercise is defined as a relative term, implying that a bout(s) of exercise is fine for some individuals while excessive for other individuals due to the differences in a variety of factors, such as physical fitness and genetics [4]. Physical fatigue, as a common physiological phenomenon during exercise, is a direct reflection of the degree of exercise. Accurate detection and evaluation of physical fatigue levels can effectively prevent excessive exercise and further reduce physical injury caused by excessive exercise.

Fatigue is a term used to describe a subjective feeling of tiredness or lack of energy. Objectively evaluating fatigue is a challenging task as a person’s subjective feelings cannot be easily assessed by other people [5]. The rating of perceived exertion (RPE) [6] is a measure of fatigue that has been widely used for fatigue assessment in sport science, specifically in running research [7,8,9]. As the RPE has advantages, such as being noninvasive, unobtrusive, noninterruptive and easy to use, many previous studies on fatigue assessment have used it as the ground truth. Moreover, previous studies [10,11] indicated that the RPE represented feedback from cardiovascular, respiratory and musculoskeletal systems and it provides an overall fatigue state assessment of a subject, while a single biomechanical or physiological parameter usually provides very limited information. So, in this study we collected the RPE of subjects at each experiment stage and used it as the physical fatigue ground truth for model training and testing.

With the development of wearable devices [12], more and more physiological parameters could be easily collected. Many researchers have been trying to use various physiological parameters collected by wearable devices to assess physical fatigue. One of the physiological signals for physical fatigue detection is an electromyogram (EMG) [13,14,15,16]. EMGs can reflect the electrical activity of local muscle, which is related to the physical fatigue of the local muscle. Physical fatigue not only reduces the body’s exercise ability but also causes neurological function decline. So, electroencephalograms (EEG) are another physiological index used for physical fatigue assessment [17,18]. Unfortunately, both EMGs and EEGs are weak bioelectric signals and are easily disturbed by many kinds of noise. Additionally, their acquisitions require professional operations to paste the acquisition electrodes to relevant body parts, so they are not widely used.

Heart rate variability (HRV), a tiny time variation between adjacent heartbeats, was demonstrated to have a relationship with autonomic nervous activities [19]. In recent years, machine learning algorithms based on HRV signals have become a research hotspot in various applications, such as noise detection [20], cuff-less blood pressure measurement [21], mental fatigue evaluation [22] and exercise-induced physical fatigue evaluation [23]. Compared with exercise-induced physical fatigue evaluation, there have been more achievements in mental fatigue evaluation. For HRV-based mental fatigue evaluation, one research team used the kernel principal component method to select important HRV features which have a strong relationship with fatigue states [24]. Their study results show that selected features can easily distinguish between normal samples and fatigue samples. Another research team concentrated on using neural networks and HRV analysis with a power spectral density algorithm to build a driver fatigue detection model, and an accuracy of 90% was achieved [25]. Moreover, decision trees, support vector machines and K-nearest neighbor classifiers have been also proposed to quantify mental fatigue [26].

As a comparison, a few recent efforts have been made towards HRV-based physical fatigue assessment. Ramos et al. [27] combined EMG features and HRV features to build a binary SVM classifier. By analyzing HRV from blood volume pulse signals, Cosoli et al. [28] evaluated the performance of two machine learning algorithms in distinguishing between nonfatigue and fatigue conditions and presented a fatigue-related index to quantify the physical fatigue. Guan et al. [29] proposed a bidirectional long- and short-term memory neural network to classify physical fatigue. The model used HRV features and inertial sensor signals as inputs and achieved 80.55% accuracy. Nevertheless, most of these models either used multiple kinds of signals or coarsely classified physical fatigue into two levels. Therefore, the purpose of this study was to investigate whether the machine learning method combined with HRV, which has been proven to be useful in mental fatigue assessment, is also effective for continuous and real-time monitoring of physical fatigue during exercise. Furthermore, the study also aimed to investigate which HRV features were the most significant in the classification. The selection and analysis of relevant features may also be important for improving the interpretability of the physical fatigue assessment model.

Based on the results of previous studies and the advancements in machine learning technology, we proposed a novel method for the automatic and objective classification of physical fatigue. First, we proposed a feature selection method to remove useless HRV features that have a low correlation with physical fatigue and redundant HRV features that have a high correlation with the selected features. Then, four machine learning algorithms were trained to classify fatigue using the selected features. Experimental results for 80 healthy subjects indicate that the model trained using the selected features could classify physical fatigue with a high accuracy of 85.5%.

The remainder of this paper is organized as follows. Section 2 introduces the data collection experiment and the physical fatigue evaluation modeling methods. Section 3 provides the physical-fatigue-related HRV feature selection results and the physical fatigue classification results. The obtained fatigue classification results with different machine learning methods are presented and discussed in Section 4. Finally, the study is concluded in Section 5.

## 2. Materials and Methods

### 2.1. Data Collection

A total of 80 healthy subjects were recruited for participation in the data collection experiments; the statistical anthropometric characteristics of all subjects are summarized in Table 1. The subjects were asked to perform a preset treadmill exercise, which was modified from the Bruce protocol [30], and during the test, their ECG data were collected. The experiment process is shown in Table 2; it started with a 5 min pre-rest, during which the subjects were asked to stand still on the treadmill. Following this was the exercise stage, during which the subjects began to run at a speed of 3 km/h, and the speed increased to the next preset value every 5 min until reaching the maximum preset speed, and the subjects would run at this maximum preset speed until they were physically exhausted. It was not necessary to reach the maximum speed during the exercise stage, and the exercise could be terminated at any time the participant signaled that he was exhausted.

**Figure 1:**
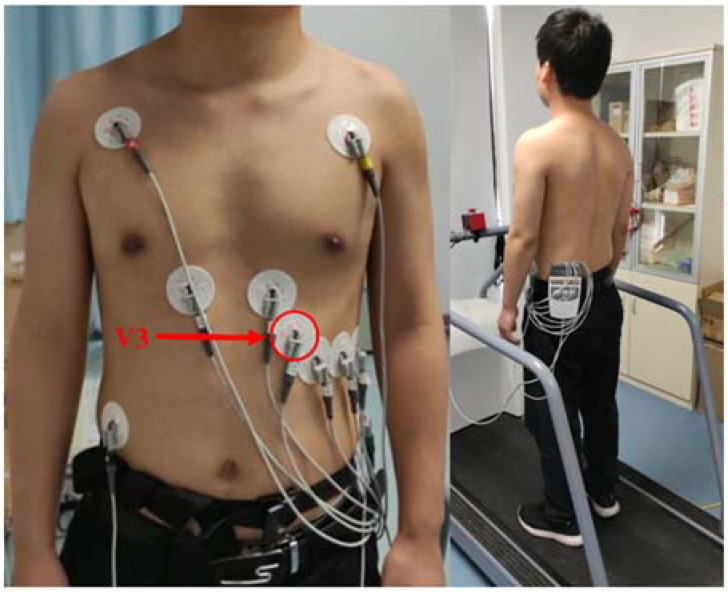
The scenario of the data collection experiment.

The data collection scenario is shown in Figure 1. The subjects were asked to wear a 12-lead ECG device (GE Medical System Information Technologies, INC, CardioSoft Cardiac Testing System). The ECG device used in our study was specially designed for exercise ECG monitoring, and hardware anti-noise design and software filtering algorithms were made to ensure the high quality of the collected exercise ECG signal. In addition, we further compared and analyzed the quality of 12-lead ECG signals, and finally selected a V3-lead ECG signal, which had the best signal quality for subsequent HRV extraction and fatigue evaluation.

The RPE scale ranged from 6 to 20, and the subject was free to choose any integer value within this range. Prior to starting the run, we explained the RPE scale listed in Table 3 to each subject. During the running experiments, the fatigue states of subjects were recorded with the RPE. At the end of each stage, the subjects were asked to report their RPE. Three classes were defined based on the RPE values: (i) “Rested” for values from 6 to 10; (ii) “A bit tired” for values from 11 to 16; and (iii) “Tired” for values from 17 to 20.

**Figure 2:**
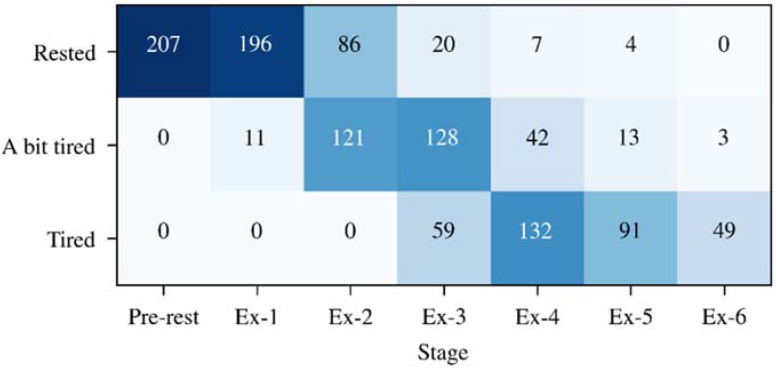
The distribution of the collected dataset.

Each participant participated in at least 1 session and at most 3 sessions (with an interval of 1 week) of data acquisition experiments. Each session lasted 20 min to 60 min, and a total of 207 sessions were collected. Figure 2 shows the fatigue state distribution of the dataset collected in the 207 sessions of experiments. It can be seen that the perception of tiredness had individual differences. Under the same exercise intensity and time, e.g., at the EX-3 stage, participants reported being “Rested” in 20 sessions, “A bit tired” in 128 sessions and “Tired” in 59 sessions. In addition, all 80 subjects finished the first 3 exercise stages (to EX-3 stage), while from the Ex-4 stage onwards, there were subjects who stopped the exercise because of exhaustion.

The study was approved by the Institutional Review Board of Shenzhen Institute of Advanced Technology, Chinese Academy of Sciences. All subjects signed their written informed consent before the experiments.

### 2.2. Preprocessing and Feature Extraction

The sampling rate of ECG data was 200 Hz. In order to eliminate electronic noise and motion artifacts, the sampled signals were preprocessed using a Butterworth low-pass filter with a cutoff frequency of 50 Hz and a nine-level wavelet decomposition with the order 8 Daubechies wavelet. As the guideline [31] recommended that the ECG records used for HRV analysis should last for at least 5 min, we segmented the raw ECG with a 5 min sliding window without overlap and extracted HRV features from each segment.

Over a specific time period, time domain features are just measurements of the mean and variability in the time interval between heartbeats, which is alternately referred to as normal-to-normal intervals (NN). The common time domain features include the mean of NN interval sequence (meanNN), the mean of heart rate sequence (meanHR), the standard deviation of NN interval sequence (SDNN) and the root mean square of successive differences in NN interval sequence (RMSSD). Another two features calculated by successive differences in NN interval sequence are the number of these differences greater than 50 ms (NN50) and the percentage of NN50 in total intervals (pNN50). By segmenting the long NN interval sequence into several nonoverlapping chunks with a chosen time window (1 min in this work), two types of HRV features, including the standard deviation of the averages of segmented chunks (SDANN) and the average of the standard deviations of segmented chunks (SDNNi), can be calculated. In addition to these statistical features, there are two geometric HRV features based on the NN interval sequence histogram with bins of 1/128 s. The HRV triangular index (HRVTi) is the ratio of the total number of all intervals to the height of the histogram. Additionally, the triangular interpolation of the histogram (TINN) is the baseline width of the minimum square difference triangular interpolation of the highest peak of the histogram.

For HRV frequency domain analysis, power spectrum density (PSD) was computed using the Lomb–Scargle method. Three main components were derived from the heart rate power spectrum, namely the very low frequency band (VLF) ranging between 0.0033 Hz and 0.04 Hz, the low = frequency band (LF) ranging between 0.04 Hz and 0.15 Hz and the high-frequency band (HF) ranging between 0.15 Hz and 0.4 Hz. The VLF component has been reported to be associated with arrhythmic death [32] and high inflammation [33]. The LF component appears to be sensitive to both sympathetic and parasympathetic activities, whereas the HF component is primarily mediated by the parasympathetic nervous activity [34]. Therefore, the LF/HF ratio has been regarded as a measure of physical workload and stress [35].

Aimed at the nonlinearity of heart rate signal, a number of nonlinear techniques have been applied to HRV analysis, which was thought to be an effective way to describe the changes in the biological signal. Three nonlinear methods were used for HRV analysis in this work, namely sample entropy (sampen), Poincare plot (SD1, SD2 and SD1/SD2) and detrended fluctuation analysis (α, α_1_ and α_2_).

Using MATLAB R2018a with the help of Physionet Cardiovascular Signal toolbox [36], a total of 24 features, including 10 time domain features, 7 frequency domain features and 7 nonlinear features, were computed for further processing. All the obtained HRV features are listed in Table 4.

### 2.3. Feature Selection

Feature selection is essential for training an effective model. There is no doubt that any unnecessary features, including unimportant features and redundant features, will increase the computational cost of model training and the risk of model overfitting, decrease the interpretability of the model and reduce the generalization performance of the model on the test set. Thus, dropping features with a weak correlation with physical fatigue (unimportant features) and removing highly redundant features are two steps needed during feature selection. The proposed feature selection method is described in Algorithm 1.
**Algorithm 1** **Feature selection.****Input:** Original features Φ, input data $\left\{(X_{1},y_{1}),\ldots ,(X_{N},y_{N})\right\}$**Output:** Selected features $\Phi^{\prime }$  1: Initialize thresholds $r_{1}$*,*
$r_{2}$  2: Initialize number of repeats T  3: **for**
f in Φ
**do**  4:     Compute actual Gini importance $I_{\left\{f\right\}}$ from $\left\{(X_{1},y_{1}),\ldots ,(X_{N},y_{N})\right\}$ according to Equation (1)  5: **end for**  6: **for**
i=1→T
**do**  7:     Shuffle the labels $y_{1},\ldots ,y_{N}$, which is referred to as $y_{1}^{\prime },\ldots ,y_{N}^{\prime }$  8:     **for**
f in Φ
**do**  9:         Compute new Gini importance of $D_{\left\{f\right\}}^{i}$ from $\left\{(X_{1},y_{1}^{\prime }),\ldots ,(X_{N},y_{N}^{\prime })\right\}$ according to Equation (1)10:     **end for**11: **end for**12: **for**
f in Φ
**do**13:     Compute score of the feature according to Equation (4), which is referred to as $S_{\left\{f\right\}}$14: **end for**15: Select the features with a score lower than $r_{1}$, which is referred to as $\Phi_{1}$16: Delete the features $\Phi_{1}$ from Φ17: Compute the correlation matrix of features Σ according to Equation (5)18: Select the features with less actual Gini importance in each pair of features with a correlation above $r_{2}$ which is referred to as $\Phi_{2}$19: Delete the features $\Phi_{2}$ from Φ20: Obtain the remaining features in Φ, which is referred to as selected features $\Phi^{\prime }$

#### 2.3.1. Dropping Unimportant Features

Dropping low-correlated features requires the scores of all the features at first. The method based on feature importance of random forest (RF) and permutation importance [37] was proposed to score the importance of features. RF provides a Gini importance for the assessment of feature importance. Suppose that we have an input dataset with N instances $\left\{(X_{1},y_{1}),\ldots ,(X_{N},y_{N})\right\}$ where each $X_{i}=\left\{x_{\left\{f_{1}\right\}},\ldots ,x_{\left\{f_{m}\right\}}\right\}$ is a vector with m features and $y_{i}$ is the corresponding label. First, RF uses the dataset to establish its model. Then, Gini importance of feature f is defined as the sum of the impurity improvement of all the nodes n in all trees S using the feature during the training phase, according to Equation (1):

(1) $$I_{\left\{f\right\}}=\underset{S}{\sum }\underset{n}{\sum }\Delta \mathrm{Gini}\left(n,S\right)$$

The decrease in Gini impurity resulting from optimal split ΔGini(n) is defined as

(2) $$\Delta \mathrm{Gini}\left(n\right)=\mathrm{Gini}\left(n\right)-p_{l}\Delta \mathrm{Gini}\left(n_{l}\right)-p_{r}\Delta \mathrm{Gini}\left(n_{r}\right)$$

(3) $$\mathrm{Gini}\left(n\right)=1-\overset{K}{\underset{k=1}{\sum }}p_{n,k}^{2}$$

where Gini(n) denotes Gini impurity at the node n; $n_{l}$ and $n_{r}$ denote the child nodes of n; $p_{l}$ and $p_{r}$ denote the ratio of the child nodes’ sample size to the total sample size; and $\;p_{n,k}$ denotes the ratio of class k={0,1,…,K} in node n.

Unlike the common permutation importance, the label rather than the feature was permuted in this method. After shuffling the labels for the first time, the new dataset can be expressed as $\{(X_{1},y_{1}^{\prime }),\ldots ,(X_{N},y_{N}^{\prime })\},$ where $y_{1}^{\prime },\ldots ,y_{N}^{\prime }$ is a permutation of the actual labels. After training the RF model with the new dataset, we could compute the Gini importance of feature f, which is referred to as $D_{\left\{f\right\}}^{1}$. By repeating T times on permutation of labels at random, the null importance distributions $D_{\left\{f\right\}}=\left\{D_{\left\{f\right\}}^{1},\ldots ,D_{\left\{f\right\}}^{T}\right\}$ of various features were created to demonstrate how the model can make sense of a feature disregarding the original labels.

Randomly reordering labels could reduce the Gini importance of all features, because the input data no longer correspond to the real labels obtained in the real world. If the model relies heavily on a feature in its prediction, its importance is particularly affected. Thus, a metric to score a feature is calculating the percentage of the feature’s null importance distribution as less than the actual importance. The formula for calculating this score of the feature f is given by Equation (4).

(4) $$\mathrm{score}=\frac{\mathrm{count}(D_{\left\{f\right\}}<I_{\left\{f\right\}})}{T}\times 100\%$$

where count() denotes counting the elements that meet the criteria, $D_{\left\{f\right\}}$ is a set of null importance distributions of f with the number of repeats of T, and $I_{\left\{f\right\}}$ is the actual Gini importance of f.

From another viewpoint, the score is a kind of quantitative index adapted from the original feature importance. Compared with the original Gini importance, the score is more effective at selecting features with high importance. The features with a score lower than the threshold $r_{1}$ will be dropped as “unimportant features”, which contribute little to the model.

#### 2.3.2. Removing Redundant Features

The Pearson correlation coefficient was implemented to compute the correlation matrix and measure the redundancy between two features. Pearson correlation coefficient r provides an indicator to quantitatively evaluate the linear correlation between two variables [38]. It has a value ranging from −1 to +1, where −1 indicates a perfect negative linear relationship, 0 indicates no linear relationship, and +1 indicates a perfect positive linear relationship. The closer the absolute value of *r* to 1, the stronger the correlation. Given two variables, *X* and *Y*, the Pearson correlation coefficient r between *X* and *Y* is defined as Equation (5):

(5) $$r=\frac{\sum_{i=1}^{N}\left(X_{i}-\bar{X}\right)\left(Y_{i}-\bar{Y}\right)}{\sqrt{\sum_{i=1}^{N}{\left(X_{i}-\bar{X}\right)}^{2}}\sqrt{\sum_{i=1}^{N}{\left(Y_{i}-\bar{Y}\right)}^{2}}}$$

where *N* is the number of the variable, $X_{i}$ and $Y_{i}$ are the values of X and Y for the i_th_ individual, and $\bar{X}$ and $\bar{Y}$ are the averages of X and Y.

In consequence, the larger the absolute value of the correlation coefficient between two features, the higher the mutual substitutability and the redundancy of the two features, and vice versa. As for each pair of features whose correlation is higher than a threshold $r_{2}$, the less important one will be regarded as the “redundant feature” and removed.

### 2.4. Physical Fatigue Classification

The last step is using machine learning algorithms to accurately classify physical fatigue levels. The classification model adopted four supervised machine learning algorithms, namely decision tree (DT), support vector machine (SVM), K-nearest neighbor (KNN) and light gradient boosting machine (LightGBM). These classification models were trained with the best features obtained by the feature selection method described in Section 2.3.

The performance metrics of the evaluation model were accuracy (ACC), precision, recall and F1 score (F1), and their definitions are listed in Equations (6)–(9)

(6) $$\mathrm{ACC}=\frac{\mathrm{TP}+\mathrm{TN}}{\mathrm{TP}+\mathrm{FP}+\mathrm{TN}+\mathrm{FN}}$$

(7) $$\mathrm{Precision}=\frac{\mathrm{TP}}{\mathrm{TP}+\mathrm{FP}}$$

(8) $$\mathrm{Recall}=\frac{\mathrm{TP}}{\mathrm{TP}+\mathrm{FN}}$$

(9) $$F1=\frac{2\times \mathrm{Precison}\times \mathrm{Recall}}{\mathrm{Precison}+\mathrm{Recall}}$$

where TP refers to the number of correctly classified samples in a certain class, FP refers to the number of samples misclassified as a certain class when they belong to other classes, TN refers to the number of correctly classified samples in other classes, and FN refers to the number of samples belonging to a certain class that was misclassified as other classes. The average of these metrics among classes was calculated to obtain a final evaluation of the model’s performance.

The 10-fold cross validation method was used to evaluate the performance of these models. In order to prevent a subject’s data from being used partly for training and partly for testing, each iteration was trained with the data of 72 subjects and tested with the data of the remaining 8 subjects. The average performance of the 10 iterations was used as the final result.

## 3. Results

### 3.1. Optimal Feature Set

**Figure 3:**
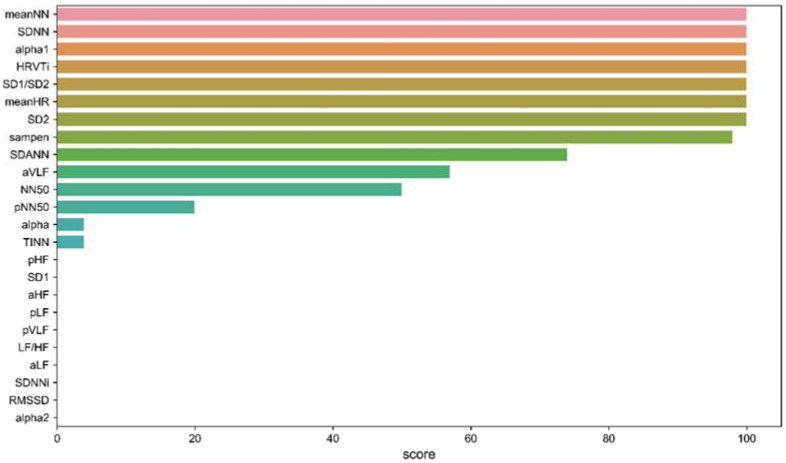
The scores calculated by Equation (4) of all the 24 original features.

Using Equation (1), the scores of the 31 original features were calculated (t = 100) and are shown in Figure 3. When setting the threshold $r_{1}$ to 0, we selected 10 unimportant features and removed them. The remaining 14 important features include meanNN, meanHR, SDNN, NN50, pNN50, SDANN, HRVTi, TINN, aVLF, sampen, SD2, SD1/SD2, α and α1.

**Figure 4:**
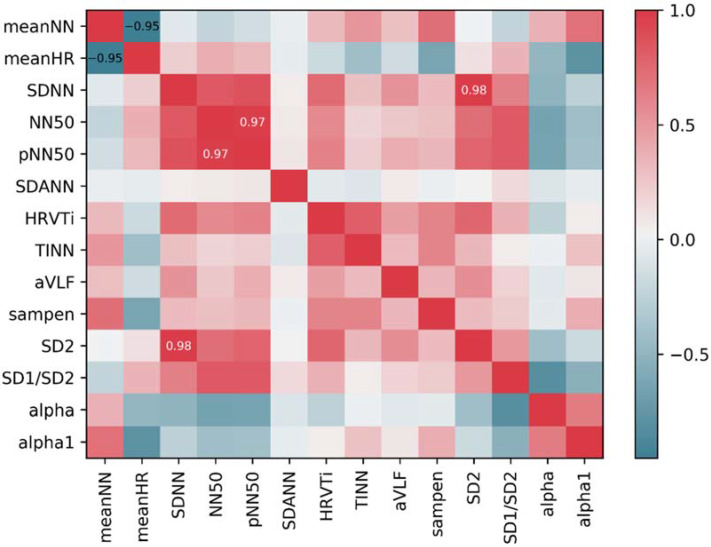
Correlations between 14 important features.

Figure 4 shows the correlations between 14 important features. As we can see, there were three pairs of features (meanNN and meanHR, pNN50 and NN50, SD2 and SDNN) with correlation magnitudes greater than the threshold *r_2_* which was set to 0.9. Then, we removed the redundant features, including meanNN, pNN50 and SDNN, which were least important features in each pair.

After the feature selection process, a total of 11 features with high importance and low redundancy were finally selected, resulting in an optimal feature set for modeling. The selected 11 features are given in Table 5.

### 3.2. Classification Performance

Table 6 shows the average accuracy, precision, recall and F1 score of four machine learning models using different features in assessing physical fatigue. On the one hand, the average performance of models using selected features was superior to the performance of models using all features. For the DT, SVM, KNN and LightGBM models trained with selected HRV features, the average F1 score increased by 6.3%, 4.0%, 2.2% and 2.0%, respectively, when compared with corresponding models trained with all HRV features. On the other hand, the standard deviations of the models trained with selected HRV features were reduced, which means the models were more stable when the selected features were used. Therefore, it can be seen that both the performance and the stability of the models were increased by the selected features, which verifies the effectiveness of our proposed feature selection method.

In addition, it was also shown that LightGBM outperformed other models in all the performance metrics, yielding an accuracy of 0.855 and an F1 score of 0.801. The performance demonstrated the possibility of using HRV for objective physical fatigue assessment.

## 4. Discussion

### 4.1. Performance Analysis

**Figure 5:**
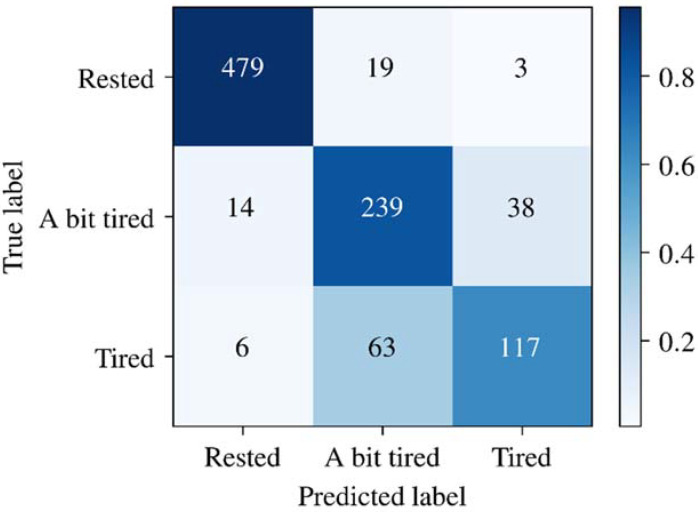
Overall confusion Matrix of LightGBM of the 10-fold cross validation.

The overall confusion matrix of LightGBM in the 10-fold cross validation is shown in Figure 5. The row labels indicate the true classes for samples in each row, while the column labels indicate the predicted classes for samples in each column. The numbers labeled in each grid show the number of samples classified into the classes labeled in the row, and the labels shown in the row are the true classes of the samples. The color represents the proportion of the aforementioned samples to all samples in the same row.

From the results shown in Figure 5, we can see that the model performed best in predicting “Rested” samples and worst in predicting “Tired” samples, indicating the model’s lack of sensitivity in discriminating “Tired” from “A bit tired”. One of the factors affecting the model performance in distinguishing between the two labels is the imbalance of the dataset. In the collected dataset, there are more rested samples than tired samples. Due to the lack of sufficient tired samples, the classifier was insufficient in describing tired samples, so the trained model had a poor performance in generalizing the “tired” label.

In addition, unlike most of the previous studies, which coarsely classify fatigue states into “Tired” and “Non-tired”, this study had one “Non-tired” state, namely “Rested”, and two levels of tiredness, namely “A bit tired” and “Tired”; as there may be bias in individual perception of the two levels of tiredness states, the classification performance for these two levels of tiredness is relatively inferior. The accuracy of the model would be greatly improved if “A bit tired” and “Tired” were merged into one category.

### 4.2. Comparison with Related Works

Our results suggest that the meanHR, NN50, SDANN, HRVTi, TINN, aVLF, sampen, SD2, SD1/SD2, α and α_1_ are the key HRV features for physical fatigue assessment. Inputting too few features or too many features may decrease the classification performance.

The HRV time domain features have been used in drivers’ sleepiness detection. Abtahi et al. [39] conducted a variance analysis between groups and found that there was a statistically significant difference for these time domain features, including meanNN, SDNN, SDANN, SDNNi and NN50. According to their results, the meanNN and SDNN increased when drivers’ mental state transformed from alert to severe sleepiness. The data analysis in [40] also showed that meanNN, SDNN and HRVTi were associated with drivers’ mental stress levels.

Previous studies have explored the frequency domain features of HRV to detect drivers’ mental fatigue. Some studies have shown that there is a significant rise in the LF/HF when the driver became drowsy [41]. While some studies suggest that the LF/HF has no significant changes when human states changed [39], other studies reported that the LF/HF even decreased with mental workload and mental stress increases [25,42]. Therefore, a study reported that the change direction and degree of HRV linear indexes may not be the same in different degrees of mental fatigue [26]. The results from our study show that the VLF was related to physical fatigue, which is consistent with the previous research results reported in [43].

The analysis in [44] showed that SD1 and SD2 decreased after a table tennis match, indicating activation of the sympathetic system and, simultaneously, deactivation of the parasympathetic system. Another study [45] pointed out that α_1_ decreased when running at low intensity. They suggested that α_1_ can provide the opportunity to track physiological status in real time to monitor exercise fatigue. Similar to the aforementioned studies, our study suggests that SD2, SD1/SD2, α and α_1_ can help to assess physical fatigue.

### 4.3. Limitations

Although we obtained promising results for LightGBM using the selected features, there were still limitations. For example, the number of subjects involved in this study was small, which may affect the stability of the models. In addition, the results of our analysis verify that heart rate is an important indicator for the final evaluation of physical fatigue. However, heart rate can be affected by many factors, e.g., diseases (including hyperthyroidism [46], diabetes [47]), emotion [48] and age [49]. Future work should be carried out to study the influence of factors affecting heart rate on the proposed physical fatigue assessment model.

## 5. Conclusions

The application of HRV and machine learning algorithms in physical fatigue assessment was studied in this paper. First, 24 HRV features of four domains were computed from the original ECG. Then, a two-step feature selection method was proposed to select the best features. After feature selection, 13 original features were removed, and 11 optimal features were selected and used as the input of the model. These selected HRV features identified for physical fatigue detection are meanHR, NN50, SDANN, HRVTi, TINN, aVLF, sampen, SD2, SD1/SD2, α and α_1_. Four machine learning algorithms, DT, SVM, KNN and LightGBM, were used to build classifiers that automatically detect the fatigue state. LightGBM achieved the best performance and had an accuracy of 0.855 and an F1 score of 0.801. The results verify the feasibility of using HRV to evaluate physical fatigue states. By using the features selected by our feature selection method, the proposed model achieved superior performance in assessing the physical fatigue state. Furthermore, the selected features can be applied to wearable ECG devices for physical fatigue assessment during exercise in real time.

In future works, more subjects with diseases (e.g, diabetes, hypertension, heart disease) and subjects with different levels of physical fitness and different ages will be included to increase the reliability of physical fatigue assessment. Finally, other machine learning or deep learning models and features based on other physiological signals would be considered.

## Figures and Tables

**Table 1:** Participants’ statistical characteristics.

Statistical Characteristic	Value
Number of Subjects	80 (42 Males, 38 Females)
Age (years)	29.1 ± 6.5
Height (cm)	168.0 ± 8.1
Weight (kg)	61.7 ± 11.2

**Table 2:** Protocol of the modified Bruce treadmill test.

Stage	Duration (min)	Speed (km/h)	Incline (%)
Pre-rest	5	0	0
Ex-1	5	3	5
Ex-2	5	5	5
Ex-3	5	6.4	5
Ex-4	5	7.8	5
Ex-5	5	10.2	5
Ex-6	Until exhausted	11.6	5

**Table 3:** The RPE scale and its description.

Borg Rating	Description
6	Nothing
7 to 8	Very, very light
9 to 10	Very light
11 to 12	Fairly light
13 to 14	Somewhat hard
15 to 16	Hard
17 to 18	Very hard
19 to 20	Very, very hard

**Table 4:** All HRV features.

Measures	Feature	Unit	Description
Time domain	meanNN	ms	Mean of NN interval sequence.
	meanHR	1/min	Mean of heart rate sequence.
	SDNN	ms	Standard deviation of NN interval sequence.
	RMSSD	ms	Root mean square of successive differences in NN interval sequence.
	NN50	count	Number of successive differences in NN interval sequences greater than 50 ms.
	pNN50	%	Percentage of NN50 in total intervals.
	SDANN	ms	Standard deviation of the averages of the segmented chunks.
	SDNNi	ms	Average of the standard deviations of the segmented chunks.
	HRVTi	-	Ratio of total number of all intervals to the height of the histogram.
	TINN	ms	Baseline width of the minimum square difference triangular interpolation of the highest peak of the histogram.
Frequency domain	aVLF	ms^2^	Absolute powers of VLF band.
	aLF	ms^2^	Absolute powers of LF band.
	aHF	ms^2^	Absolute powers of HF band.
	LF/HF	-	Ratio of aLF/aHF.
	peakVLF	Hz	Peak frequency for VLF band.
	peakLF	Hz	Peak frequency for LF band.
	peakHF	Hz	Peak frequency for HF band.
Nonlinear domain	sampen	-	Negative natural logarithm of the conditional probability that two sequences remain similar at the next point.
	SD1	ms	Standard deviations along the major axis of the ellipse.
	SD2	ms	Standard deviations along the minor axis of the ellipse.
	SD1/SD2	-	Ratio of SD1 to SD2.
	α	-	Slope of a fitting line of the root mean square fluctuation of an integrated and detrended time series on a log–log scale.
	α_1_	-	α on first linear region.
	α_2_	-	α on second linear region.

**Table 5:** Optimal feature set.

Time Domain	Frequency Domain	Nonlinear Domain
meanHR	aVLF	sampen
NN50		SD2
SDANN		SD1/SD2
HRVTi		α
TINN		α_1_

**Table 6:** Performance of the four machine learning models using different features.

Model	Using All Features				Using Selected Features			
	Accuracy	Precision	Recall	F1 Score	Accuracy	Precision	Recall	F1 Score
DT	0.728 ± 0.043	0.646 ± 0.062	0.644 ± 0.053	0.642 ± 0.056	0.772 ± 0.030	0.711 ± 0.036	0.706 ± 0.034	0.705 ± 0.034
KNN	0.780 ± 0.045	0.712 ± 0.044	0.694 ± 0.041	0.696 ± 0.044	0.805 ± 0.020	0.754 ± 0.031	0.735 ± 0.038	0.736 ± 0.033
SVM	0.810 ± 0.038	0.752 ± 0.048	0.748 ± 0.053	0.747 ± 0.052	0.831 ± 0.037	0.780 ± 0.045	0.770 ± 0.040	0.769 ± 0.043
LightGBM	0.841 ± 0.030	0.811 ± 0.035	0.777 ± 0.041	0.781 ± 0.038	0.855 ± 0.015	0.829 ± 0.032	0.800 ± 0.031	0.801 ± 0.025

## Data Availability

The data used in this study are not publicly available because the institutional review board did not grant permission, but they are available from the corresponding author upon reasonable request.
